# Integrative Ranking of Enhancer Networks Facilitates the Discovery of Epigenetic Markers in Cancer

**DOI:** 10.3389/fgene.2021.664654

**Published:** 2021-05-31

**Authors:** Qi Wang, Yonghe Wu, Tim Vorberg, Roland Eils, Carl Herrmann

**Affiliations:** ^1^Health Data Science Unit, Medical Faculty Heidelberg and BioQuant, Heidelberg, Germany; ^2^Faculty of Biosciences, Heidelberg University, Heidelberg, Germany; ^3^Division of Molecular Genetics, German Cancer Research Center (DKFZ), Heidelberg, Germany; ^4^Digital Health Center, Berlin Institute of Health (BIH) and Charité, Berlin, Germany

**Keywords:** enhancer, epigenetics, histone modification, chromatin interaction, network analysis

## Abstract

Regulation of gene expression through multiple epigenetic components is a highly combinatorial process. Alterations in any of these layers, as is commonly found in cancer diseases, can lead to a cascade of downstream effects on tumor suppressor or oncogenes. Hence, deciphering the effects of epigenetic alterations on regulatory elements requires innovative computational approaches that can benefit from the huge amounts of epigenomic datasets that are available from multiple consortia, such as Roadmap or BluePrint. We developed a software tool named IRENE (Integrative Ranking of Epigenetic Network of Enhancers), which performs quantitative analyses on differential epigenetic modifications through an integrated, network-based approach. The method takes into account the additive effect of alterations on multiple regulatory elements of a gene. Applying this tool to well-characterized test cases, it successfully found many known cancer genes from publicly available cancer epigenome datasets.

## Introduction

Epigenetic alterations are frequent in many cancers. In particular, DNA methylation and histone modifications are two main mechanisms that allow cancer cells to alter transcription without changing the DNA sequences, and lead to many abnormalities such as persistent activation of cell cycle control genes or deactivation of DNA repair genes. For example, promoter DNA hypo-methylation accompanied by histone hyper-acetylation is frequently observed in the activation of oncogenes in cancer. Besides, aberrant activation of distal regulatory elements is often associated with the up-regulation of cancer-promoting genes. Interestingly, epigenetic modifications at proximal and distal regulatory elements often appear to be earlier events than the gene expression (Hartley et al., 2013; Ziller et al., 2014), and can hence serve as potential early markers in cancer diagnosis.

Various histone modifications on promoters have been categorized into either activation or repression effects on gene expression. Such effects can be measured by comparing histone alteration levels between tumor and their corresponding normal tissues using ChIP-Seq (Karlic et al., 2010). A number of tools, such as ChIPComp (Chen et al., 2015), ChIPDiff (Xu et al., 2008), ChIPnorm (Nair et al., 2012), csaw (Lun and Smyth, 2015), DBChIP (Liang and Keles, 2012), DiffBind (Stark and Brown, 2011), MAnorm (Shao et al., 2012), RSEG (Song and Smith, 2011) have demonstrated their usefulness in cancer studies by comparing the histone intensities between two conditions (see Steinhauser et al., 2016 for a review of these tools). However, they are limited to the comparison of a single histone mark. Furthermore, many tools such as jMOSAiCS (Zeng et al., 2013), IDEAS (Zhang et al., 2016), and ChromHMM (Ernst and Kellis, 2012) are able to perform integrative analyses across multiple epigenetic marks. However, while these tools provide an integrated description of the epigenetic characteristics at individual genome loci, they do not take into account the combined effects of these changes at multiple regulatory elements controlling a gene.

As previously mentioned, many histone modifications that potentially regulate gene expression also occur in other genomic regions besides promoters. Enhancers are distal regulatory elements that interact with gene promoters through chromosomal loops to regulate gene transcription. Most of the enhancers are located within ±1 Mb of the transcription start site (TSS) of their target genes (Maston et al., 2006). Enhancer activity is regulated through epigenetic modifications (Zentner et al., 2011), including positive regulation from histone marks, such as H3K27ac (Creyghton et al., 2010; Stasevich et al., 2014) and H3K4me1 (Heintzman et al., 2007; Calo and Wysocka, 2013), and negative regulation by H3K27me3 (Charlet et al., 2016) and H3K9me3 (Zhu et al., 2012).

**Figure 1:**
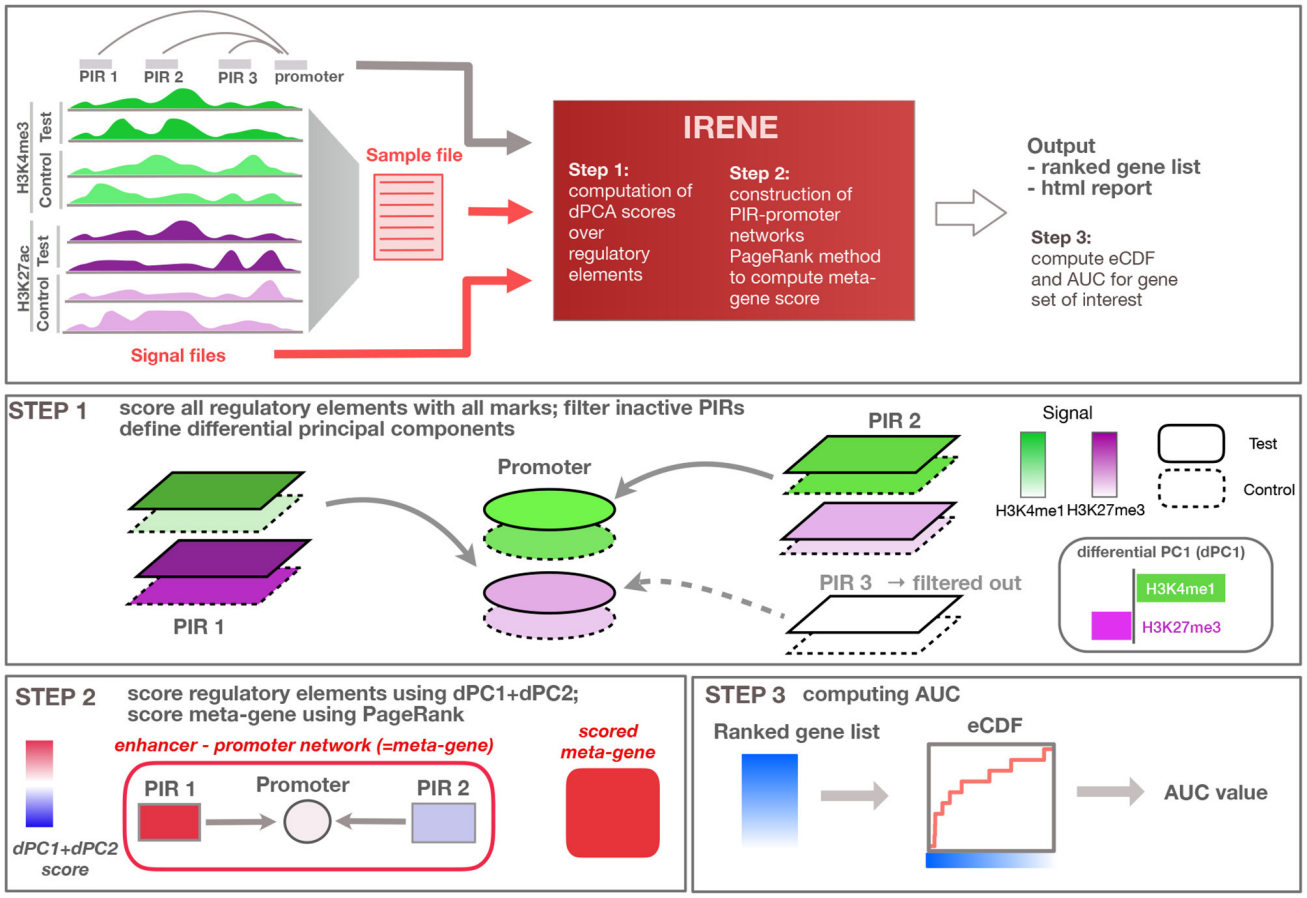
Overview of the method. General overview of the input/output of the IRENE method; user defined input is indicated in red, while input provided by the method (e.g., regulatory loci and interactions) are displayed in gray. Step 1: Regulatory elements are scored using all epigenetic modifications, and related to the target gene. Step 2: Epigenetic alterations are scored using the first dPC and combined using PageRank into an integrated meta-gene score. Step 3: Ranked gene lists based on the score are converted to eCDF curves showing the enrichment of a given gene set within the top-ranked genes, and corresponding area under the curve (AUC) values are computed.

Given the complexity of epigenetic regulation, novel tools are required to combine this information, and create a comprehensive overview of the differential epigenetic landscape, integrating multiple data layers. The method we developed, named IRENE (Integrative ranking with an epigenetic network of enhancers), combines a quantitative analysis on multiple differential epigenetic modifications with an integrated, network-based approach, in which we integrated two levels of epigenetic information: the signal intensity of each epigenetic mark, and the relationships between promoters and distal regulatory elements known as enhancers (Figure 1). In this paper, we describe the method and present the test cases. In our benchmarking tests on cancer datasets, the IRENE ranked lists have higher relevance to cancer marker genes (CMGs) than the other approaches. Being implemented as an R package, IRENE is an easy to use method allowing gene ranking between two conditions and highlighting potential cancer biomarkers.

## Results

###  IRENE: Epigenetic Ranking With an Epigenetic Network of Enhancers

IRENE analyzes epigenetic changes between two biological conditions (e.g., ChIP-seq data for histone modifications or whole-genome bisulfite sequencing for DNA methylation), and translates the differential signals at multiple regulatory elements into a unique score (Figure 1). Hence, IRENE performs a double integration, both across multiple epigenetic datasets and across different regulatory regions linked to a gene. To integrate multiple datasets, we use dPCA, which captures the directions of the greatest differential variance comparing two conditions, at each regulatory element (see section Materials and Methods) (Ji et al., 2013). As the goal of our method is to capture the differential signal at proximal and distal regulatory elements, we performed a dPCA analysis both at gene promoters and distal regulatory elements, which we call promoter interacting regions (PIRs) extracted from the 4DGenome database (Teng et al., 2015). Similar to standard PCA, differential PCA captures the directions of the greatest differential variance along several differential principal components (dPCs). We selected the first two dPCs, which appear to capture the differential signal both from activating and repressive epigenetic marks. The sum of the absolute values of dPC1 and dPC2 at each regulatory element was used as a score for this element. These scores are summarized as a weighted network relating regulatory elements to their target genes. The network consists of promoters and connected PIRs. Oriented edges from PIRs to promoters indicate a 3D interaction between these regulatory elements. Despite being in principle a bipartite graph (with nodes being either PIRs or promoters), we do not make a distinction between these two types of regulatory elements. A random walk based method then assigns a score to the corresponding gene. The output of the method is a ranked list of genes from the most to the least affected one, which incorporates both promoter and enhancer alterations. As a comparison, we also generated ranked lists based only on the promoter score (named promoter ranked lists in the following), discarding the contributions from distal PIR elements. This approach can be applied whenever two conditions are to be compared, for example, normal/tumor tissue, various tumor subtypes, or different developmental stages. More details are given in the Materials and Methods section. In order to benchmark our method, we used seven test cases consisting of tumor samples for seven different tumor types and normal matching samples. For each of these test cases, we compiled a list of CMGs (Supplementary Table 2) from the literature, and considered tissue-specific genes (TSGs) obtained from the ArchS4 database (Lachmann et al., 2018) as controls.

###  Cancer Marker Genes Are Scored Higher by Incorporating Enhancer in the Ranking

**Figure 2:**
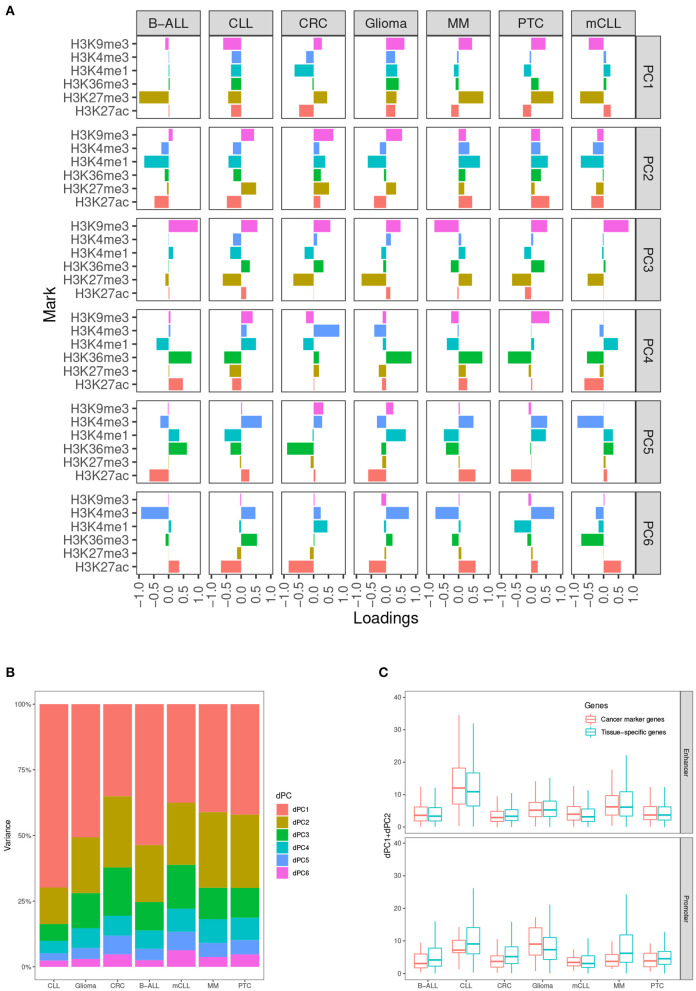
Differential principal components. **(A)** Contributions of the six histone marks to each differential principal component (dPC). **(B)** Variances accounted for each dPC in the seven test cases. **(C)** Values of dPC1+dPC2 in the seven test cases, comparing cancer marker genes (CMGs) with TSGs, both for enhancers (top), and promoters (bottom).

In our analysis, we determined that taking into account the first two dPCs is able to capture most of the differential variance for both activating and repressive epigenetic modifications (Figures 2A,B). After comparing the dPC1+dPC2 values between the CMGs and TSGs in each test case, we found that the scores from CMGs are generally higher than the scores of the TSGs for the enhancers, whereas the situation is less clear at promoters. This might indicate that most of the differential signal between tumor and normal occurs at distal regulatory regions. (Figure 2C).

**Figure 3:**
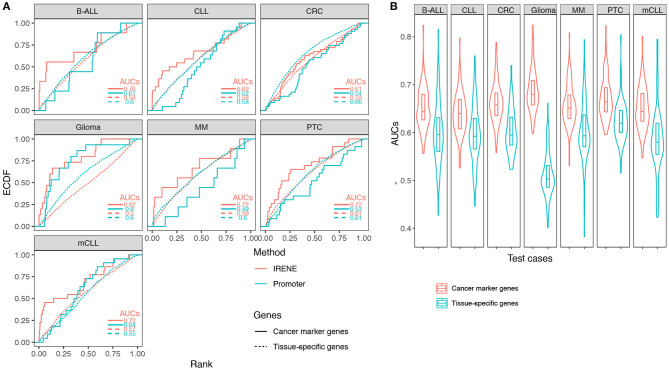
**(A)** Empirical cumulative density function (ECDF) curves regarding the cancer marker genes and tissue-specific genes in seven test cases. The marker gene ranks using IRENE scores (red) are compared against their ranks using the promoter scores (cyan). **(B)** Distribution of the area under the curve (AUC) values using the cancer marker gene (CMG) sets from CancerMine in the seven test cases, compared to randomly picked tissue-specific genes to define equal size sets.

Using the ranked gene lists generated by IRENE, we further computed the area under the curve (AUC) for the empirical cumulative density function (ECDF) of the high-confidence CMG ranks as a benchmarking approach, as described in the methods. First, we examined the IRENE ranks computed using the dPC1+dPC2 on gene promoters and their targeting enhancers, and found that the marker genes are ranked higher than TSGs in every test case, indicating that our approach captures the specific differential epigenetic signals at CMGs (Figure 3A). Moreover, both for CMGs and TSGs, the IRENE AUC values are higher than the AUC values computed using the dPC1+dPC2 of gene promoters only (Figure 3A). The fact that the genes ranked higher in IRENE suggests that a significant part of the altered epigenetic alteration arises from distal enhancer regions. We then validated these findings on the larger CMG and TSG gene sets, and we found the AUCs of CMGs are all significantly higher (one-tailed *t*-test **p**-value < 0.01) than the AUCs of TSGs (Figure 3B).

**Figure 4:**
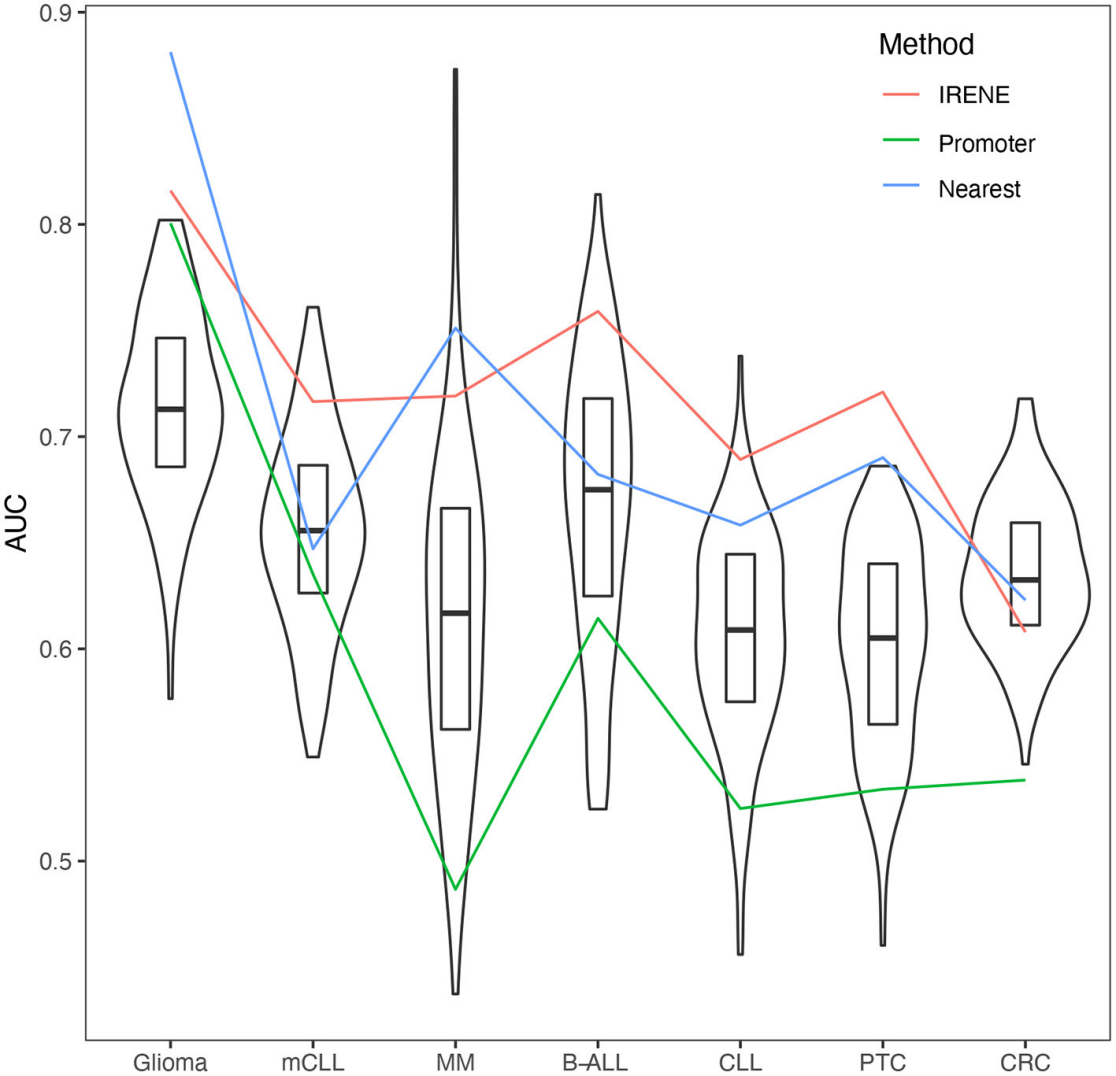
Area under the curves (AUCs) of empirical cumulative density function (ECDF) curves of dPC1+dPC2 ranks from randomized promoter–enhancer interactions. The boxplots indicate the 25–75% quantile ranges from benchmarking each cancer marker gene set with 1,000 different rewired promoter–enhancer networks, whereas the red lines show the AUCs with the original promoter–enhancer interactions from IRENE using experimentally detected interactions (red), and interactions assigned by the nearest promoters (blue), and only promoters (green) rank lists.

Some genes have a much high number of linked enhancers than others. To test whether this might bias the ranks of these genes, we performed 1,000 degree-preserving random perturbations, which completely rewired the enhancer–promoter graph but maintaining the degree distribution. We used the high-confidence CMGs in the benchmarking, and the AUCs with randomly assigned enhancers dropped 5–10% on average, indicating that the higher ranks of CMGs are not explained by their higher connectivity (Figure 4).

We compared the target gene assignment provided by the 4DGenome database, which is based on experimental evidence, with the simpler nearest-gene assignment. As can be observed in Figure 4, both approaches lead to comparable results, in line with recent reports indicating that the nearest gene assignment is reasonably effective in linking enhancers with target genes (Moore et al., 2020).

As mentioned in the Introduction, several other methods have been developed to integrate multiple epigenetic marks over genomic regions. Most of these methods provide qualitative analysis in the form of discrete chromatin states. To our knowledge, none of these methods apply a network-based integration as in IRENE to summarize regulatory elements related to the same gene. In order to provide a comparison, we focused on one of the mostly used such method, ChromHMM, which integrates various histone marks into discrete chromatin states (Ernst and Kellis, 2012). We combined ChromHMM with the Chromswitch method (Jessa and Kleinman, 2018), which computes a differential score between two groups of samples over specific regions. Applying this scoring approach to promoter regions, we compared the ranked lists obtained by IRENE at promoter regions with the ChromHMM-based ranks for the Glioma/normal brain test case, and found that the AUC values of the CMGs related to Glioma are significantly higher for the IRENE method (Supplementary Figure 2).

###  Network Analyses Characterized the Highly Ranked Genes in the IRENE and Promoter List

**Figure 5:**
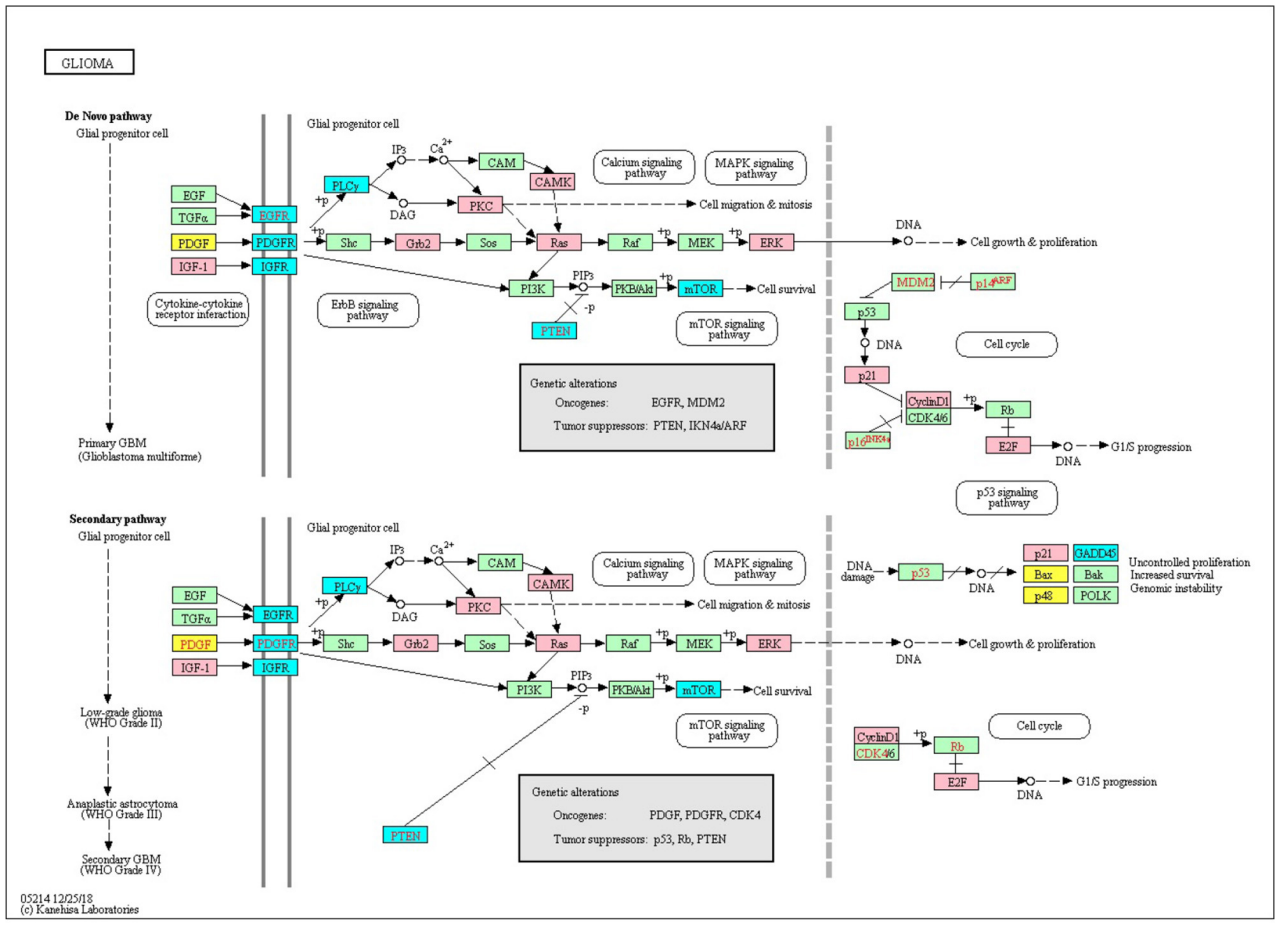
The top 25% genes from the IRENE and promoter rank list are highlighted on the KEGG glioma signaling pathway. Pink, genes from the IRENE list; yellow, genes from the promoter list; cyan, genes from both lists.

We downloaded 184 KEGG pathways in KGML format and loaded them as directed graphs using KEGGgraph (Zhang and Wiemann, 2009). Then we took the top 15% genes from the IRENE and promoter rank lists in each one of the seven test cases, and mapped the genes to the KEGG cancer signaling pathway (hsa05200). In total, the reference pathway contains 531 genes and 1989 interactions, and on average 208 of the 531 genes are found in the IRENE rank lists, while only 152 genes are found in the promoter rank lists. In addition, the IRENE-ranked genes differ from promoter-ranked genes in both in-degrees and out-degrees of the nodes (Table 1). As the IRENE nodes generally have higher in-degrees than out-degrees in the graph presentation of the reference pathway, implying the IRENE genes are more often targeted by the other regulatory genes on their enhancers as they harbor more differential enhancers. We further examined the glioma signaling pathway (hsa05214) and found 19 genes from the IRENE rank list and 10 genes from the promoter rank list in the glioma test case (Figure 5). One common gene, *EGFR*, is in both lists and has been reported to undergo tight control through epigenetic regulation on both promoters and enhancers (McInerney et al., 2000; Liu et al., 2015; Jameson et al., 2019). Moreover, nine genes are present only in the IRENE rank list, such as *CCND1*, which has been reported to be regulated by an estrogen-mediated enhancer (Eeckhoute et al., 2006). In conclusion, this analysis shows that the IRENE methods provide a ranked gene list, which is enriched for high-ranking, cancer-relevant genes.

**Table 1:** Graph properties in respect of the nodes from the IRENE and promoter rank lists.

	**Node number**		**Median in-degree**		**Median out-degree**	
	**IRENE**	**Promoter**	**IRENE**	**Promoter**	**IRENE**	**Promoter**
CLL	214	167	2	2	1	3
Glioma	193	133	2	1	1	1
CRC	219	168	2	0	1	3
B-ALL	180	124	1	1	1	0
mCLL	211	168	2	0	1	3
MM	219	165	2	1	1	1
PTC	219	137	2	1	1	3

## Discussion

**Figure 6:**
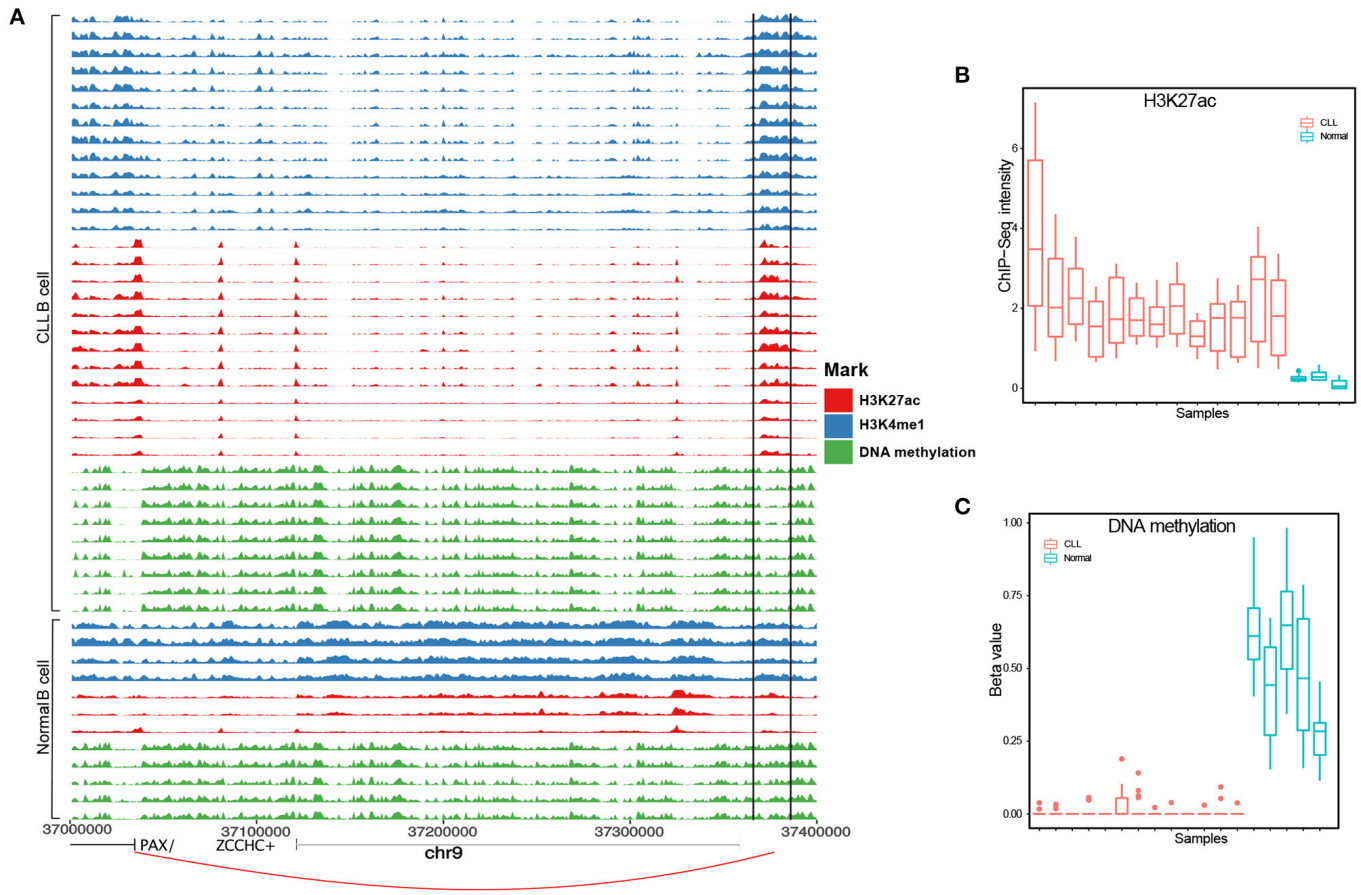
A known PAX5 enhancer (chr9:37,370,916-37,371, and 635) in CLL exhibits hyperacetylation and hypomethylation [analysis of variance (ANOVA) *p*-value <0.05]. **(A)** PAX5 enhancer positions in each track are surrounded by two black solid lines. **(B)** H3K27ac and **(C)** DNA methylation levels between CLL and healthy samples.

From the above benchmarking on seven cancer test case studies, we showed that IRENE is a more comprehensive approach comparing to the current frequently used approaches such as separate ranking gene promoters and enhancers. This highlights the importance of epigenetic regulation through distant enhancer regions. Using IRENE, users cannot only discover the genes which show significantly epigenetic alterations on their promoters, but also the ones that are connected with strong epigenetic modifications on distal interacting enhancers, which facilitates the discovery of potential epigenetic marker genes. On the other hand, by interpreting the higher ranked genes mapped to the existing pathways, the user may also find the enhancers of interests from their differential epigenetic modifications. For example, we found the *PAX5* gene to have a significantly higher rank in the IRENE list compared to the promoter-only list in the two CLL case studies, which implies that *PAX5* is extensively regulated by enhancers. *PAX5* is a key transcription factor in B-cell development, and its promoters have no significant epigenetic alterations in the CLL case studies. However, this gene is associated with several hyperacetylated and hypomethylated distal enhancers, one of which is located at 330 kilobases (kb) upstream of the *PAX5* TSS, and has been also found as extensively mutated in CLL (Puente et al., 2015) (Figure 6). The deletion of this enhancer resulted in a 40% reduction in the expression of *PAX5* expression and chromatin interaction of this enhancer and *PAX5* has been proven from chromosome conformation capture sequencing (4C-Seq) analysis (Puente et al., 2015). The main difficulty of this study is obtaining cell type specific enhancer–promoter interactions, as the high-resolution chromatin interaction map for the cancer cells is currently not available. We have tested two alternative approaches in this study, using either the experimentally validated chromatin interaction or distance-based interactions. The performance of the above two approaches are similar (Figure 4). We believe better performance can be achieved when cell type specific enhancer–promoter interactions are available in the future, and using IRENE, user can replace the interaction map with a more specific one when applicable. Being a differential approach comparing two conditions, it might be affected by the possible heterogeneity of the groups being compared. If the heterogeneity is due to biological reasons (for example, different subtypes in the disease group), the comparison will be affected by the greater variance within one group. However, if the heterogeneity is of technical nature, then this noise will likely be buffered by the fact that our method integrates multiple regions to score the genes.

## Conclusions

Genome-wide integrative epigenetic analysis is challenging and essential in many comparative studies. As far as we know, IRENE is the first tool that integrates quantitative and genome context information in the differential epigenetic analysis. Applying this tool to well-characterized test cases, it detects a number of candidate genes with significant epigenetic alterations, and comprehensive benchmarking validated these findings in cancer studies. As epigenomic datasets accumulate, the computational approaches employed in this study would be highly relevant in both comparative and integrative analysis of the epigenetic landscape. The discovery of novel epigenetic targets in cancers not only unfolds the fundamental mechanisms in tumorigenesis and development but also serves as an emerging resource for molecular diagnosis and treatment.

## Materials and Methods

### Data Preparation

#### Retrieving Epigenetic Modification and Chromatin Interaction Datasets

Genome-wide ChIP-seq data are downloaded in BigWig format from NIH Roadmap Epigenomics (Bernstein et al., 2010), Blueprint (Adams et al., 2012), and the International Human Epigenome Consortium (IHEC) (Stunnenberg et al., 2016). We selected the six most frequently studied histone marks: H3K27ac, H3K27me3, H3K36me3, H3K4me1, H3K4me3, and H3K9me3. These resources allow us to investigate the histone modification differences between tumor and normal tissues (Supplementary Table 1). For restricting the comparisons to the genomic loci of interests (promoters and enhancers), we downloaded the GRCh37 and GRCh38 coordinates of promoters from the eukaryotic promoter database (EPD) (Dreos et al., 2013), and the promoter interacting regions (PIRs) from the 4DGenome database (Teng et al., 2015). We treated the PIRs as potential enhancer regions, and filtered for tissue-specific enhancers by requiring the presence of H3K4me1 or H3K27ac peaks (peak calls provided in the Supplementary Table 1) in at least two samples from either tumor or normal tissues. By doing this, we enrich for cell type specific PIRs, which show a tissue-driven clustering (Supplementary Figure 1). The promoter coordinates were extended to ±1000 base pairs around the original coordinates. The sum of the numeric values from the BigWig blocks which overlap with the promoter and interacting regions are available from our project homepage. To build the relationships between and enhancers and promoters, we also download all the experimentally validated chromatin interaction datasets in various human tissues from 4DGenome.

#### Defining Disease and Control Datasets

We used histone modification datasets from seven cancer types in this study, i.e., B-ALL, CRC, glioma, MM, PTC, CLL, and mCLL from the Blueprint and IHEC consortia. For each cancer dataset, we paired it with the available dataset from the healthy tissue from which the cancer is most likely originated from. For example, the B-ALL, CLL, and MM were all compared against the healthy B cells in our design (see Supplementary Table 1 for the pairs of normal/tumors used).

#### Definition of Cancer Marker Genes and Tissue-Specific Genes

We evaluated our algorithm on a small set of high-confidence CMGs, which is based on the tier-1 genes of the corresponding tissues from the Cancer Gene Consensus (CGC-t1) (Sondka et al., 2018) (Supplementary Table 2). As a negative control, we compiled a list of tissue-specific genes (TSGs) related to the tissues of interest for the tumor cases from ARCHS4_Tissues (https://maayanlab.cloud/archs4/). There are 2,318 genes for every tissue in the list. To validate our findings on independent, larger datasets of CMGs and TSGs, we compiled additional CMG lists containing 4,212 CMGs from 90 different cancer types from CancerMine (Lever et al., 2019), which incorporates the manual curated lists including the Cancer Gene Consensus (Sondka et al., 2018) and IntOGen (Gonzalez-Perez et al., 2013).

### Data Processing Procedures

#### Combining Histone Marks

The epigenetic intensities on regulatory elements were summarized on a 1 kb scale, then power-transformed and quantile normalized. We use the dPCA (Ji et al., 2013) to decompose the matrix *D* representing the difference between *M* epigenetic datasets at *G* genomic loci comparing two groups of samples, into matrices *B* and *V* (1)

(1) $$\begin{array}{r} D_{G\times M}=B_{G\times R}V_{R\times M}+E \end{array}$$

where *E* is the random sampling noise.

We use the first *k* dPCs to represent the major changes between two conditions. We implemented an R wrapper function for dPCA in our tool, which takes the mean differences of the normalized ChIP-Seq signals in each genomic locus between two biological conditions as input, and returns the dPCs from dPCA. The definition of dPCs varies between the test cases (Figure 2A). The largest variances of the positive and negative histone mark components are captured by dPC1 and dPC2 in our test case studies (Figure 2B). Therefore, we selected the sum of the absolute values of the first two dPCs for representing the overall differences of these epigenetic marks.

#### Promoter–Enhancer Interaction Analyses

In our approach, the enhancer–promoter relationships are described as a weighted bipartite graph, in which both enhancers and promoters are represented as vertices, and edges are directed from enhancers to their target promoters (Figure 1 Step 1). The weights of the vertices are defined as the sum of the absolute values of the first two dPCs when combining multiple epigenetic marks, or the absolute value of the difference if a single epigenetic mark is considered. We adopt an algorithm called “PageRank,” which is originally designed for evaluating the importance of web pages (Brin and Page, 1998), for ranking the magnitude of epigenetic alterations in each gene. We use the “personalized” PageRank implemented in igraph (Rye et al., 2011) to summarize the weights of one promoter and its connected enhancers into a unique meta-gene score (Figure 1 Step 2). Since our enhancer–promoter network is a directed graph, all the enhancer weights will eventually be attributed to their target promoter using PageRank, yielding a unified score for each gene, which can be used to rank the genes. Overall, there are ~251, 000 promoter interacting fragments in the promoter–enhancer interaction networks in our case studies, which is 8.5 times the number of promoters in the networks. The number of the interacting fragments targeting a gene varies from none to 227, and on average, 21 interacting fragments are targeting a promoter in the networks.

#### Scoring Ranked Lists

Using the gene ranks computed as described in the previous section, we can now evaluate the enrichment of a specific gene set G in the ranked list by computing the empirical cumulative distribution function (ECDF) obtained ranking the genes in decreasing order based on the previously described rank, and summing the indicator function

(2) $$eCDF_{G}(k)=\sum_{i=1}^{k}\delta_{i}\;with\;\;\delta_{i}=\left\{ \begin{array}{l} 1\;if\;g_{i}\in \;G\\ 0\;if\;g_{i}\notin \;G \end{array}\right.$$

We use the area under the curve (AUC) as a measure of the enrichment of the gene set G, with AUC = 0.5 corresponding to a random distribution of the genes in G inside the ranked list.

#### Comparison With ChromHMM

We applied the ChromHMM method (version v1.22) to the Glioma and the healthy brain control samples (see Supplementary Table 1). The 6 histone marks were integrated into 10 chromatin states, of which 2 correspond to active promoter regions and one to active enhancer regions (Supplementary Figure 2B). The chromswitch package (Jessa and Kleinman, 2018) (v. 1.12.0) from Bioconductor was applied to the promoter and PIR regions linked to promoters for specific chromatin states. The chromswitch method determines a consensus score between changes occurring in chromatin state within a group of sample, and the labels of these samples. Hence, a maximal consensus score for a region of interest would correspond to changes in a chromatin state within the region of interest occurring only in the samples of one of the two groups. A minimal consensus score would on the opposite correspond to changes in chromatin states in the region of interest occurring in samples, which are randomly distributed over the two groups. For each gene, we compute a score by averaging the consensus score of all regulatory elements related to this gene, and use this score to rank the genes, as a comparison to the IRENE ranking.

## Data Availability Statement

The R package is available at https://github.com/hdsu-bioquant/irene. The datasets generated for this study can be found in the https://github.com/hdsu-bioquant/ irene-data. We also designed a web interface that allows users to trace back the epigenetic alterations of every enhancer and promoter, as well as every sample which is used for computing the score. We use Rmarkdown to generate static HTML pages and created a web site for presenting the results from our test case studies, which can also be found under the project home page. Users may also take advantage of this function to create a report that highlights a few genes of their interests and share the studies with the audience.

## Author Contributions

CH designed and supervised this project. QW and CH drafted the manuscript. QW wrote and tested the software. YW and TV participated in software testing. YW, TV, and RE revised the manuscript. All authors contributed to the article and approved the submitted version.

## Conflict of Interest

The authors declare that the research was conducted in the absence of any commercial or financial relationships that could be construed as a potential conflict of interest.
