# Supplementary material for: Integrative Ranking of Enhancer Networks Facilitates the Discovery of Epigenetic Markers in Cancer
Source: Front Genet. 2021 May 31;12:664654. doi: 10.3389/fgene.2021.664654 (PMC8201988; doi:10.3389/fgene.2021.664654)
Supplement: Supplementary file 1 [file Data_Sheet_1.zip › Data Sheet 1 (1).PDF]

Supplementary Material

1 SUPPLEMENTARY TABLES AND FIGURES

1.1 Figures

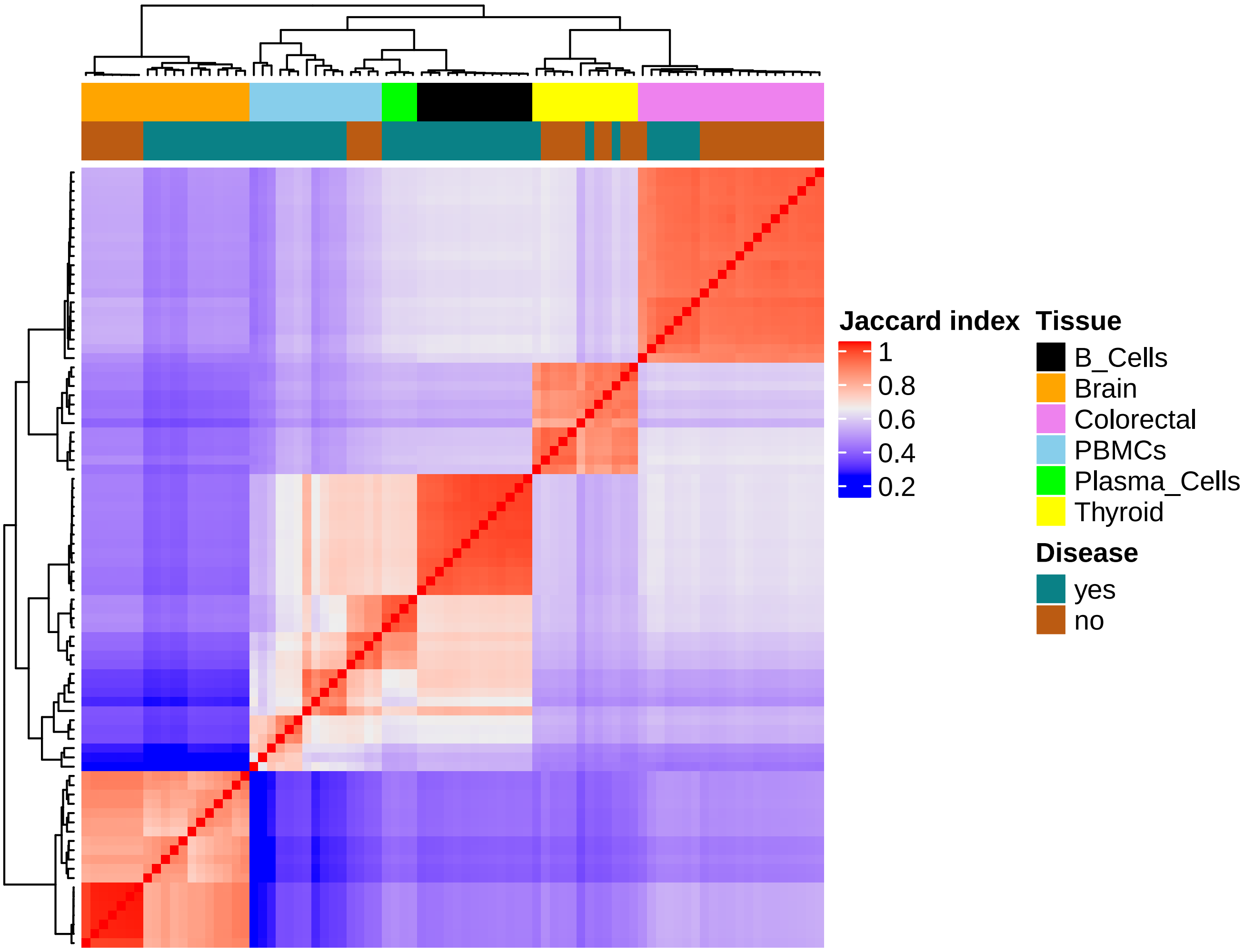

Figure S1. Clustering of common PIRs between samples shows enrichment of cell-type specificity. We intersect the peaks obtained from enhancers marks (H3K4me1 and/or H3K27ac) in each sample with the list of PIRs (see "Data preparation" section), and computed the Jaccard index using the intersection and union of filtered PIRs between samples.

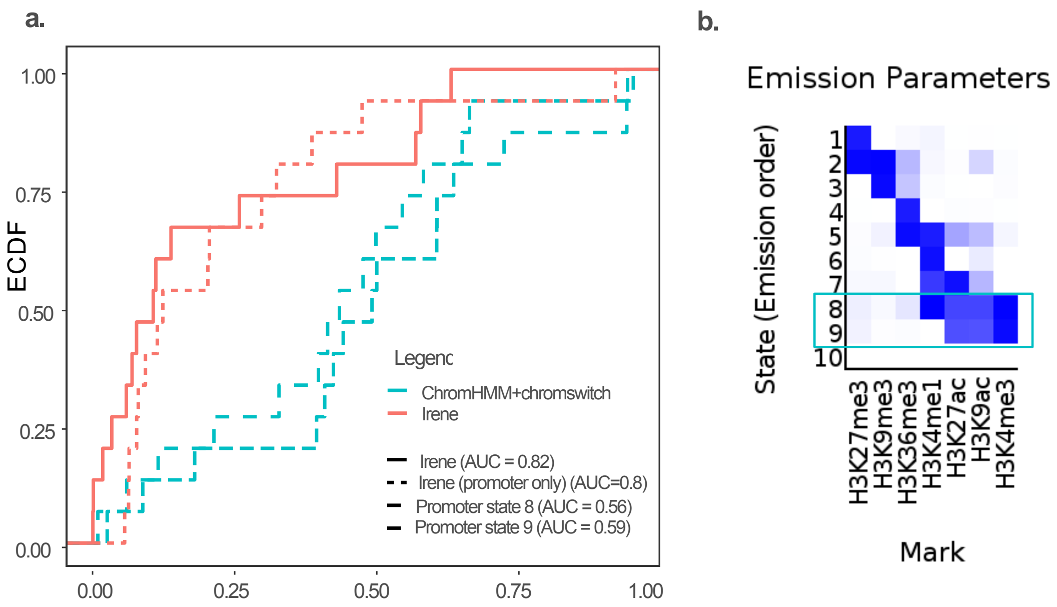

Figure S2. Comparison with ChromHMM based method on GLioma case (a) eCDF curves for CMG in the Glioma case, comparing various approaches: ranking computed by IRENE (with promoters and PIRs, full red line), IRENE only using promoters (dotted red line), the ChromHMM+chromswitch method for the promoter state 8 and promomter state 9 (blue dashed lines). (b) ChromHMM 10-states learned from the Glioma/normal brain samples, with states 8/9 (=promoter states) highlighted.
